# Diabetes-related weight change in a Canadian First Nation cohort

**DOI:** 10.1080/22423982.2017.1340548

**Published:** 2017-06-22

**Authors:** ND Riediger, V Lukianchuk, J Roulette, LM Lix, L Elliott, SG Bruce

**Affiliations:** ^a^ Department of Community Health Sciences, Rady Faculty of Health Sciences, University of Manitoba, Winnipeg, Manitoba, Canada; ^b^ Ongomiizwin – Research, Indigenous Institute of Health and Healing, Rady Faculty of Health Sciences, University of Manitoba, Winnipeg, Manitoba, Canada; ^c^ Department of Human Nutritional Sciences, Faculty of Agriculture and Food Sciences, University of Manitoba, Winnipeg, Manitoba, Canada; ^d^ Sandy Bay First Nation Health Centre, Sandy Bay Ojibway First Nation, Manitoba, Canada; ^e^ Centre for Healthcare Innovation, University of Manitoba, Winnipeg, Manitoba, Canada

**Keywords:** Diabetes, weight gain, weight loss, obesity, First Nation, metformin

## Abstract

The Canadian First Nations population is disproportionately burdened by diabetes and diabetes complications. Body weight management is purported to be important in the prevention and management of diabetes. In this study, we sought to describe weight change in a First Nation cohort according to diabetes status and management. Study data were from two diabetes screening studies in Sandy Bay First Nation in Manitoba, Canada, collected in 2002/2003 (baseline) and 2011/2012 (follow-up). The cohort was composed of respondents to both screening studies (n=171). Fasting blood samples, anthropometric, health and demographic data were collected. At baseline, 24.8% (n=41) of the cohort members had diabetes. At follow-up, an additional 20.6% (n=34) developed diabetes. Among all participants with diabetes (long-term and incident cases), 66.6% lost weight between the two study periods. Among only participants with long-term diabetes (>8 years), 31.7% lost >10 kg. HbA1c at baseline, positive change in HbA1c over time, and use of metformin were significantly associated with weight loss ≥5%, independent of age, sex, and BMI at baseline. Further research is needed to better understand if and how diabetes-related weight loss contributes to morbidity and mortality in this First Nation population.

## Introduction

Canadian First Nations populations have a disproportionate burden of diabetes compared with the non-First Nations population [1,2]. We have reported a sex- and age-standardised prevalence of diabetes at 39% in 2011/2012 in the study community, Sandy Bay Ojibway First Nation [3], which is not significantly different as compared with 2002/2003. Acute measures of glucose metabolism (mean fasting glucose, HbA1c) were significantly higher among participants without diabetes in 2011/2012, indicating an adverse shift in health status among otherwise healthy people. Furthermore, we reported that over half of new cases of diabetes in the community are among individuals less than 40 years old [4]. Therefore the study community represents a population with a particularly high burden of disease.

There is also a high prevalence of obesity among First Nations populations [5,6] that is likely driving diabetes rates. However, we have recently reported that the prevalence of obesity in the study population was significantly lower in in 2011/2012 at 48.7% compared with 60.8% in 2002/2003 [3]. These findings suggests that further investigation into weight change using a longitudinal design in the study population is warranted, particularly since prevalence of diabetes did not mirror this reduction in obesity prevalence. Previous research has shown that weight gain is a risk factor for incident diabetes [7] and weight loss has been shown to delay new onset diabetes among high-risk individuals [8,9]. However, neither body mass index (BMI) nor waist circumference were found to be significant independent predictors of incident diabetes in this population [10]. Therefore, describing changes in weight as they relate to diabetes, in a population at high risk, may contribute to broader understandings of primary or secondary prevention of diabetes complications as well as the obesity paradox. The obesity paradox refers to the higher mortality among people with type 2 diabetes of normal weight compared with those that are overweight and/or obese [11]. While this paradox may be attributed to a variety of factors, an important consideration must be the weight changes associated with diabetes diagnosis and progression. Therefore the purpose of this study is to describe weight change in a First Nation cohort with a high prevalence and incidence of diabetes, according to diabetes status and its management.

## Methods

The study community is Sandy Bay Ojibway First Nation, located in southwest Manitoba, approximately 200 km northwest of Winnipeg. This community has all-weather road access. The total on-reserve population in 2011 was approximately 4,100 people, with 50% under 19 years old. The study was approached using a community-based participatory approach [4]. Community partners described rapid weight loss among people with diabetes in the community, particularly for men, and were interested in investigating this phenomenon further.

All adults, 18 years and over and non-pregnant, were invited to participate in a Diabetes Screening Study in 2002/2003 (convenience sample) [3]. Participants had to be a registered member of Sandy Bay First Nation or a registered member of another First Nation but living in Sandy Bay. Recruitment was conducted through advertisement at the Community Health Centre, word of mouth, and home visits from community research assistants. A total of 478 community members participated, representing 44% of the eligible population. In 2011/2012 a second Diabetes Screening Study was completed with 596 participants, representing 28% of the eligible population [4]. Targeted recruitment was employed to optimise the longitudinal sample size. Specifically, we identified participants from 2002/2003 who were known to be alive and residing in the community; community research assistants then personally invited those participants to return for the 2011/2012 study through home visits. Two of the three community research assistants who were also Elders were able to identify those who had passed away or moved. Participants from both the 2002/2003 and 2011/2012 sample were linked to create an 8-year follow-up cohort. The study was approved by the University of Manitoba Health Research Ethics Board (H2011:171)

Of 478 participants from 2002/2003, 171 returned in 2011/2012, comprising 36% of the original sample. The cohort sample (n=171) was not significantly different compared with the baseline sample (n=478) in 2002/2003 according to sex, education, employment, diabetes, hypertension or obesity [4]. For example, 37% of participants without diabetes at baseline returned at follow-up, which is similar to the overall response rate at 36%. Notably, the cohort was 2 years younger compared with the baseline sample, which was mostly accounted for by fewer participants older than 50 years at baseline.

In both 2002/2003 and 2011/2012, venous blood samples were drawn by a registered nurse after a minimum 12-hour fast, processed on site at the Health Centre, and stored at −20°C. Details of the biochemical analysis have been previously described [3,12]. In the present study, fasting glucose and HbA1c measures were utilised. 2 blood pressure readings were taken with an automated blood pressure monitor after a 5 minute rest, both initially and between readings, and averaged. Anthropometric measures, weight, height and waist circumference were taken by trained research assistants using established methods previously described [3,12].

Participants’ medical histories, including previous diagnosis of diabetes or hypertension, as well as medication use, were obtained by research assistant administered questionnaire. For example, participants were asked directly if they had diabetes, for how long, if they were taking any medications for their diabetes, and if so, what they were. The questionnaire also captured socioeconomic characteristics, including employment and highest level of education. Employment included any work for pay (either part-time or full-time). Highest level of education was dichotomised based on median split in the 2002/2003 sample, as < grade 9 completion and ≥ grade 9 completion [3].

BMI was calculated as weight (kg)/height (m)^2^. Obesity is defined as a BMI ≥30 kg/m^2^. Weight change was dichotomised; weight loss was defined as, BMI at follow-up minus BMI at baseline <0 and weight gain/stable weight was defined as, BMI at follow-up minus BMI at baseline ≥0. Magnitude of weight loss was determined by the change in body weight over time and categorised as >0 kg, >5 kg, and >10 kg, based on similar cut-offs used by others [13–15]. Percent body weight change was dichotomised as weight loss >5% from baseline and ≤5% [16]. Change in waist circumference was calculated as waist circumference at follow-up minus waist circumference at baseline. Diabetes is defined as currently on an oral hypoglycaemic, self-declared, or fasting glucose ≥ 7.0 mmol/L [12]. Length of time with diabetes, or diabetes status, is categorised as no diabetes, diabetes ≤8 years (incident diabetes), and diabetes >8 years (i.e. diabetes at baseline). Glucose control was measured using change in HbA1c, calculated as HbA1c at follow-up minus HbA1c at baseline. Hypertension is defined as a previous diagnosis of hypertension; or systolic blood pressure >140 mm Hg or diastolic blood pressure >90 mm Hg; or for those with diabetes, SBP ≥130 mm Hg or DBP ≥80 mm Hg [17]. Albuminuria includes both proteinuria (>1 g/L) and microalbuminuria as previously defined [18].

### Analyses

First, the percent of the sample experiencing weight loss (or stable weight/weight gain) was described according to age group, sex, employment, education, diabetes, hypertension, obesity and albuminuria at baseline. Age groups are defined as 18–29, 30–39, 40–49, and 50 years and older. Differences in weight change according to the previously listed variables were tested using a χ^2^ test of independence. All statistical analyses were conducted using the current version of SPSS (version 22) with significance set at α=0.05.

Weight loss, as well as magnitude of weight loss and percent weight change, and change in waist circumference was described according to length of time with diabetes for both men and women. Chi-square tests were used to test for differences in percent of participants losing any weight and losing >10 kg according to length of time with diabetes among men and women separately. Kruskal–Wallis tests were used to test for differences in change in waist circumference according to length of time with diabetes. Weight loss >5% from baseline was also described according to use of oral hypoglycaemic agent and diabetes status. Finally, we conducted a multivariable logistic regression model predicting percent weight loss >5% from baseline, with HbA1c at baseline, change in HbA1c, and use of oral hypoglycaemia agent as predictors; the model was also adjusted for age, sex, hypertension and BMI at baseline. Percent weight loss >5% was selected as the outcome based on the research by Kocarnik and colleagues [16], particularly in the context of including oral hypoglycaemic agents as predictors of weight loss.

## Results

Data on weight change were available for 165 of the 171 individuals who participated in both screening studies. Six individuals had missing data for weight, in either time period, for multiple reasons including partial foot or below the knee limb amputation. Nearly half the sample (n=82) lost weight over 8 years of follow-up. At baseline (i.e. 2002/2003), 24.8% (n=41) of the cohort members had diabetes (>8 years). At follow-up (i.e. 2011/2012), an additional 20.6% (n=34) developed diabetes (incident cases). Older participants and participants with diabetes, hypertension and obesity at baseline were significantly more likely to experience weight loss (Table 1). Sex, employment, highest level of education and albuminuria at baseline were not significantly associated with weight change.

**Table 1.** Description of study sample at baseline according to weight change status.

	Weight loss (n = 82)	Stable/weight gain (n = 83)	
Variables at baseline	n (%)	n (%)	p-value
Age groups			
18–29 years	12 (25.0)	36 (75.0)	0.001
30–39 years	36 (57.1)	27 (42.9)	
40–49 years	25 (61.0)	16 (39.0)	
≥50 years	9 (69.2)	4 (30.8)	
Sex			
Men	39 (50.0)	39 (50.0)	0.941
Women	43 (49.4)	44 (50.6)	
Highest level of education^a^			
< grade 9	44 (54.3)	37 (45.7)	0.209
≥ grade 9	36 (44.4)	45 (55.6)	
Employed			
Yes	21 (53.8)	18 (46.2)	0.553
No	61 (48.4)	65 (51.6)	
Diabetes			
Yes	30 (73.2)	11 (26.8)	0.001
No	52 (41.9)	72 (58.1)	
Hypertension			
Yes	40 (65.6)	21 (34.4)	0.002
No	42 (40.8)	61 (59.2)	
Obese			
Yes	57 (60.6)	37 (39.4)	0.001
No	25 (35.2)	46 (64.8)	
Albuminuria			
Yes	19/31	69 (53.1)	0.151
No	61/130	12 (38.7)	

Data presented as n (%) with p-values computed using the χ-square test of independence.^a^Refers to p-values.

Weight loss was greater among individuals with long-term diabetes. In sex-stratified analysis, magnitude of weight loss was associated with diabetes status for both men and women, based on chi-square test (Table 2). Among men with diabetes (including incident and long-term diabetes), 73% lost weight and 35% lost >10 kg. According to chi-square tests, men with both incident and long-term diabetes were significantly more likely to lose weight (p<0.001) and significantly more likely to lose more than 10 kg (p=0.002) as compared with men without diabetes. There was not a significant difference in the proportion of men that lost weight or lost >10 kg between those with incident and long-term diabetes (p=0.609). Among women with both incident and long-term diabetes, 61% lost weight and 32% of women with long-term diabetes lost >10 kg. Among women, length of time with diabetes was associated with weight loss (p=0.035) and weight loss >10 kg (p=0.001). In comparing incident and long-term diabetes cases, women did not experience significantly different risk of any weight loss. However, women with long-term diabetes were significantly more likely to lose >10 kg compared with women with incident diabetes (p=0.036). Results were similar with percent weight loss, such that both participants with incident and long-term diabetes, of both sexes, were significantly more likely to lose >5% of their body weight (Table 2). Using a Kruskal–Wallis test, among men and women combined (p=0.03), and men alone (p=0.003), diabetes status was associated with significantly different change in waist circumference; change in waist circumference was not significantly different according to diabetes status among women alone (p=0.128)

**Table 2.** Magnitude of weight loss among men and women according to diabetes status (n (%)).

Sex	Measure	No diabetes	Incident diabetes	Long-term diabetes^a^
Men	Weight loss			
	>0 kg	12 (28.6)	10 (66.7)	17 (77.3)
	>5 kg	7 (16.7)	7 (46.7)	15 (68.2)
	>10 kg	2 (4.8)	6 (40.0)	7 (31.8)
	Percent weight loss			
	≤5%	34 (81.0)	9 (47.4)	7 (38.9)
	>5%	8 (19.0)	10 (52.6)	11 (61.1)
	Change in waist circumference, cm (median (IQR))	7.25 (0.50, 10.25)	0.00 (−6.00, 4.00)	−2.00 (−4.75, 6.38)
Women	Weight loss			
	>0 kg	19 (38.8)	9 (47.4)	14 (73.7)
	>5 kg	11 (22.4)	5 (26.3)	11 (57.9)
	>10 kg	1 (2.0)	1 (5.3)	6 (31.6)
	Percent weight loss			
	≤5%	37 (74.0)	13 (56.5)	6 (42.9)
	>5%	13 (26.0)	10 (43.5)	8 (57.1)
	Change in waist circumference, cm (median (IQR))	6.25 (−5.63, 13.88)	3.75 (−1.25, 10.50)	−2.50 (−8.00, 4.50)

IQR, interquartile range

^a^Long-term diabetes is defined as >8 years.

The most common oral hypoglycaemic agent used was metformin (n=36), followed by glyburide (n=11). All participants receiving glyburide also reported receiving metformin. Use of metformin and/or glyburide was significantly associated with weight loss >5% (Chi-square test, p<0.001). Among participants with diabetes at follow-up, 35.0% (n=13) of participants NOT on metformin or glyburide lost >5% body weight, 72% (n=18) of participants on metformin alone lost >5% body weight, and 72% (n=8) of participants on both metformin and glyburide lost >5% body weight.

In multivariable logistic regression, HbA1c at baseline, change in HbA1c, and metformin use were positively and significantly associated with weight loss >5%, independent of age at baseline, sex, and BMI at baseline (Table 3). Specifically, use of metformin was associated with 3.5 times higher odds of weight loss >5% compared with someone not receiving metformin. Given the small number of participants receiving glyburide or any other oral hypoglycaemic agent, we did not disaggregate medication groups further in this analysis. An individual with an increase of 2% (ex. from 6% to 8%) in HbA1c over time was 2.5 times more likely to have lost >5% body weight compared with someone that did not experience any increase in HbA1c over time. Neither hypertension at baseline or follow-up were significantly associated with weight loss >5%, and was removed from the model.

**Table 3.** Multivariable model predicting percent weight loss ≥5% among Canadian First Nation cohort (n=165).

Variable	Odd ratio (95% confidence interval)	p-value
Age at baseline	1.029 (0.986, 1.073)	0.188
Sex		
Men	Reference	
Women	0.993 (0.449, 2.198)	0.993
BMI at baseline	1.035 (0.974, 1.100)	0.269
HbA1c at baseline	1.328 (1.011, 1.744)	0.041
Change in HbA1c	1.281 (1.012, 1.621)	0.040
Medication		
No metformin	Reference	
Metformin	3.456 (1.362, 8.774)	0.009

## Discussion

Weight loss occurred among half the Sandy Bay First Nation cohort. A significantly higher proportion of individuals with diabetes lost weight. This finding may partially explain the lower prevalence of obesity reported in 2011/2012 in the study community compared with 2002/2003 despite no significant change in prevalence of diabetes [3]. Weight gain was highest among participants 18–29 years old compared with all other age groups and was similar between men and women. The finding that young adults in the study sample were most likely to experience weight gain is consistent with the literature [19–23]. There are also age-related changes in body composition that should be taken into account in the interpretation of this age association. Unfortunately, data on body composition are not available for the present study.

Both men and women with diabetes were more likely to lose weight compared with participants without diabetes. Among participants with long-term diabetes (>8 years), 32% of men and women lost >10 kg. Among men with new cases of diabetes, 67% lost weight and 40% lost >10 kg. These results are congruent with anecdotal reports from the community regarding the pattern of rapid weight loss among men newly diagnosed with diabetes. Our multivariable model indicates that weight loss may be enhanced by use of metformin and uncontrolled glucose. Metformin has been shown to be associated with weight loss of approximately 1–3 kg following initiation and after 5 years of follow-up [24,25]. However, we have reported that metformin use was associated with loss >5% of initial body weight, granted, the present study occurred over a longer follow-up. This result is concerning given that weight loss >5% following initiation of metformin has been associated with significantly higher odds of 5-year mortality [16].

Uncontrolled glucose, specifically HbA1c at baseline and change in HbA1c, was also independently associated with weight loss >5%. Given this finding, we suspect that for many participants this weight loss was unintentional, since intentional weight loss has been associated with reductions in HbA1c [26]. Furthermore, there were no differences in diabetes prevention programming or primary care management in the community during the study time period. Notably, we have previously reported that glucose control among individuals with diabetes did not improve at the population level in 2011/2012 compared with 2002/2003 [3]. Physiological explanations for the reported weight losses include large increases in blood glucose in combination with decreases in fasting insulin that may have resulted in significant glucosuria; and/or uncontrolled glucose that may have contributed to autonomic dysfunction of the digestive system or diabetic gastroparesis, leading to reduced food intake [27]. Further adding support for weight loss being potentially unintentional is that despite nearly half of women with incident diabetes losing weight, median change in waist circumference indicated a gain in waist circumference. This suggests that weight loss did not translate to reductions in waist circumference. However, this apparently paradoxical finding may also be explained by age-related changes in body composition; typically, individuals experience a loss of fat-free mass and gain in body fat with age [19].

The community context related to food insecurity is also an important consideration in interpreting these findings. We have previously reported a high burden of food insecurity in the community, which is complicated by large distance to grocery stores, transportation barriers and food sharing with family [28]. While statistical analysis related to food security is beyond the scope of this paper, weight loss must be interpreted within this context as glucose management is likely complicated by potential fluctuations in hunger.

Our findings may point to gaps in health service provision for people in Sandy Bay. Sandy Bay has a Health Centre whose mandate is public health services including immunisation, communicable diseases and health education. Consistent primary care is not available in the community; the nearest primary care providers are a 1 hour drive away. That the magnitude of weight loss among men was not significantly different between those newly diagnosed and those with longstanding diabetes may be an indicator of late diagnosis. We have previously reported on the impact of late diagnosis in relation to hypertension [6]. A recent evaluation of Health Centre programmes supports the impact of health funding models on community health. Specifically, we found that the Health Centre does not have sufficient resources or capacity to effectively implement diabetes treatment and prevention services [29].

While controversial, the wider body of literature seems to suggest weight maintenance may be better for long-term health as compared with weight loss, which is difficult to sustain and could result in weight cycling [22,30]. Despite the emphasis in public health on optimal body weight for disease prevention, there is relatively little research on weight loss, particularly intentional versus unintentional, among those with diabetes. This gap is particularly pronounced among First Nation populations due to few longitudinal studies. Gregg and colleagues [31] reported that 45% of overweight American individuals with diabetes experienced a weight loss (>1 lb) during 9 years of follow-up, compared with 67% over 8.2 years in the present study. Compared with those with stable weight, Gregg and colleagues also report that those who lost weight were younger, more likely to be women and more likely to have been diagnosed with diabetes more recently. In contrast, we did not find a sex difference in weight loss, and weight loss was more common among older individuals.

There is a considerable amount of literature supporting a high risk of cardiovascular outcomes and cardiovascular-related mortality for individuals experiencing unintentional weight losses. Gregg and colleagues [31] report a 22% and 40% higher mortality rate among those who lost any weight and those losing at least 20 lbs (regardless of intention) compared with those having no weight change, respectively, independent of age, sex, BMI, smoking and education. When specifying further by weight loss intention, it was found that compared with those with stable weight or weight gain and not trying to lose weight, those with unintentional weight loss of any amount had a 58% higher mortality rate. Stevens and colleagues [32] also report that a weight loss >3% in 3 years was associated with an increased coronary heart disease risk (hazard ratio: 1.46) and stroke risk (hazard ratio: 1.45), indicating that even a short-term weight loss could be a sign of a looming cardiovascular event.

The present study results potentially support findings relating to the obesity paradox among individuals with diabetes [11]. It is possible that normal-weight individuals with diabetes are more likely to have experienced weight loss (likely unintentional) as compared with obese individuals with diabetes, related to uncontrolled glucose or diabetic gastroparesis, as speculated in the present study. However, more research is needed to confirm the cause of weight loss in the study population, to what extent the weight loss is unintentional, and how weight loss may be associated with cardiovascular outcomes. Given the reported relationship of weight loss among individuals taking metformin in the study community and the associated mortality with weight loss following metformin use [16], increased monitoring of weight loss following initiation of oral hypoglycaemic agents in the community may be prudent.

This study is limited by the small sample size due to a high number lost to follow-up. The results may also be influenced by underlying conditions that were not captured in the data, multiple medication use that was too complex and with too small a sample to incorporate in the analysis as well as substantially impacted by variations in adherence among the cohort. While the study sample is limited to individuals from one First Nation community, the results may provide indications of weight change patterns among other Canadian First Nations populations with high prevalence of diabetes.

## Conclusions

The high degree of weight loss among individuals with diabetes could be considered an optimistic finding given the demonstrated role that lifestyle intervention for weight loss has in reducing diabetes risk. However, the cause of weight loss is unclear in the present study and may be unintentional, which carries a higher cardiovascular risk. Based on the present findings and the prevalence of diabetes in the study community, with the goal of preventing secondary complications, interventions focussing on health-related behaviours such as diet and exercise rather than weight loss specifically may be more prudent. While weight loss can be an important part of diabetes management, it cannot be prioritised above lifestyle changes to the extent that adverse weight changes not only go unnoticed but are potentially considered beneficial. Additional research is needed to confirm intentionality of weight loss and to what extent weight may be associated with adverse outcomes.
